# Analysis of endoplasmic reticulum stress-related gene signature for the prognosis and pattern in diffuse large B cell lymphoma

**DOI:** 10.1038/s41598-023-38568-x

**Published:** 2023-08-25

**Authors:** Chaofeng Zhang, Qi Lin, Chaoqi Li, Zhimin Chen, Mengmeng Deng, Huixin Weng, Xiongpeng Zhu

**Affiliations:** 1 Department of Hematology and Rheumatology, The Affiliated Hospital of Putian University, Putian, Fujian Province China; 2 The School of Clinical Medicine, Fujian Medical University, Fuzhou, Fujian Province China; 3 Department of Pharmacy, The Affiliated Hospital of Putian University, Putian, Fujian Province China; 4 Pharmaceutical and Medical Technology College, Putian University, Putian, Fujian Province China; 5 Department of Nephrology, Blood Purification Research Center, the First Affiliated Hospital, Fujian Medical University, Fuzhou, Fujian Province China; 6 Department of Haematology, Quanzhou First Hospital of Affiliated to Fujian Medical University, Quanzhou, Fujian Province China

**Keywords:** Computational biology and bioinformatics, Data mining, Cancer, Haematological cancer

## Abstract

Diffuse large B-cell lymphoma (DLBCL) is the most common lymphoma in adults. This study aimed to determine the prognostic significance of endoplasmic reticulum (ER) stress-related genes in DLBCL. ER stress-related genes were obtained from the molecular signatures database. Gene expression data and clinical outcomes from the gene expression omnibus and TCGA datasets were collected, and differentially expressed genes (DEGs) were screened out. Gene ontology enrichment analysis, the kyoto encyclopaedia of genes and genomes pathway analysis, and geneset enrichment analysis were used to analyse the possible biological function of ER stress-related DEGs in DLBCL. Protein–protein interaction network construction using the STRING online and hub genes were identified by cytoHubba on Cytoscape software. The significant prognosis-related genes were screened, and the differential expression was validated. The immune microenvironment assessment of significant genes were evaluated. Next, the nomogram was built using univariate and multivariate Cox regression analysis. 26 ER stress-related DEGs were screened. Functional enrichment analysis showed them to be involved in the regulation of the endoplasmic reticulum mainly. *NUPR1* and *TRIB3* were identified as the most significant prognostic-related genes by comparison with the GSE10846, GSE11318, and TCGA datasets. *NUPR1* was correlated with a good prognosis and immune infiltration in DLBCL; on the other hand, high expression of *TRIB3* significantly correlated with a poor prognosis, which was an independent prognostic factor for DLBCL. In summary, we identified *NUPR1* and *TRIB3* as critical ER stress-related genes in DLBCL. NUPR1 might be involved in immune infiltration in DLBCL, and TRIB3 might serve as a potential therapeutic target and prognostic factor in DLBCL.

## Introduction

Diffuse large B-cell lymphoma (DLBCL) is the most common lymphoid neoplasm, representing approximately 30% of non-Hodgkin lymphoma (NHL)^1,2^, and it is a highly heterogeneous, aggressive disease^2,3^. Currently, R-CHOP (rituximab, cyclophosphamide, doxorubicin, vincristine, and prednisone) is the standard first-line treatment for DLBCL^2,4^, however, about 30–40% of DLBCL patients remain resistant to RCHOP and are refractory or develop relapsed^5,6^, and only 10% of patients with refractory or relapsed DLBCL could be treated using salvage immunochemotherapy followed by autologous stem cell transplantation^3,7^, implying a significant unmet medical need^8,9^. The cell-of-origin (COO) classifications^9,10^ and the International Prognostic Index (IPI) score have often been considered the two most commonly prognostic factors for patients with DLBCL^3,11,12^. However, these prognostic factors do not fully explain risk stratification, clinical outcomes in DLBCL patients^13,14^, and there is an urgent need for valuable biomarkers to guide prognostic factors and therapeutic approaches for DLBCL.

Endoplasmic reticulum (ER) stress, a state in which the unfolded and misfolded protein accumulation affects the normal physiological function of cells, refers to the excessive stress caused by dysfunction of ER stress^15,16^. Notably, ER stress signaling is associated with the development of several cancers, including DLBCL^15–17^. The unfolded protein response (UPR), controlled general translation, misfolded protein degradation, and folding enzyme production are some of the adaptive responses that cells may initiate in response to ER stress. Protein kinase RNA (PKR)-like ER kinase (PERK), activating transcription factor 6 (ATF6), and inositol-requiring enzyme 1 (IRE1) are the three types of signal transducers evolved to be involved in the regulation of UPR network^18,19^. Malignant and stromal cells have their ER homeostasis disrupted by the hostile milieu created by a confluence of oncogenic, transcriptional, and metabolic aberrations in numerous tumor forms^18,20,21^. The alterations induce a state of persistent ER stress, which has been shown to regulate several protumor characteristics in cancer cells while dynamically altering the function of innate and adaptive immune cells. Generally, aberrant activation of ER stress sensors and their downstream signalling pathways have been identified as important regulators of cancer development, metastasis, and response to chemotherapy, targeted treatments, and immunotherapy^19–23^. But the significance of ER stress-related genes in the biological features and clinical prognosis of DLBCL has not been thoroughly investigated.

In our study, we aimed to better investigate and comprehend the predictive and prognostic value of ER stress-related genes in DLBCL using public databases. First, differentially expressed genes (DEGs) were compared in DLBCL patients with normal tissues. Second, the biological activities and potential pathways of ER stress-related DEGs were validated. Third, the prognostic model and immune infiltration assessment were performed. We explored and verified to illustrate the prognostic role and biological functions of ER stress-related genes in DLBCL at the bioinformatic and experimental levels.

## Materials and methods

### Data downloading and preprocessing

DLBCL datasets (GSE56315^24^, GSE10846^25^, and GSE11318^26^) were obtained from the Gene Expression Omnibus (GEO) (http://www.ncbi.nlm.nih.gov/geo/). All three datasets were based on the GPL570 platform and came from Homo sapiens. There were 55 DLBCL patients and 33 noncancerous tissues (NCs) in the GSE56315 dataset, and all of them were included in this analysis. After removing patients with incomplete survival information, 414 DLBCL samples in the GSE10846 dataset and 200 DLBCL samples in the GSE11318 dataset were enrolled. And 48 DLBCL samples (TCGA_DLBC) in The Cancer Genome Atlas (TCGA) were downloaded from The University of California Santa Cruz (UCSC) Xena browser. A total of 295 ER stress-related geneset were obtained from the Molecular Signature Database v7.0 (MSigDB)^27^ after removing the overlapped genes (Supplementary Table S1).

### DEGs screening and gene function analysis

DEGs had been screened between DLBCL samples and lymphocytal data from normal human tonsils, which came from the GSE56315 dataset, and were explored using the limma package^28^. Two thresholds were set to determine the degree of DEGs: the adjusted *p* value < 0.05 and |log2FC|> 1. And these DEGs were intersected with the ER stress-related genesets to extract the differentially ER stress-related DEGs. For the exploration of the function of the extracted genes, the gene ontology (GO) enrichment analysis^29^, and kyoto encyclopedia of genes and genomes (KEGG) pathway analysis^30^ were conducted using the clusterProfiler package^31^. In order to evaluate the gene expression level of potential signaling pathways and biological functions, the gene set enrichment analysis (GSEA)^32^ was conducted using the “c2.cp.kegg.v6.2.-symbols” geneset, and false discovery rate (FDR) q-value ≤ 0.25 was considered as statistically significant.

### Protein–protein interaction network construction

For searching for the relationship between proteins of interest, a Protein–protein interaction (PPI) network was constructed through the Search Tool for the Retrieval of Interacting Genes (STRING; http://string-db.org)^33^ online, and the combined score was greater than 0.4 considered statistically significant. Cytoscape (version 3.7.2, http://www.cytoscape.org)^34^ was used to visualize this PPI network, and the cytoHubba plug-in Cytoscape^35^ was used to calculate the hub nodes to select the top DEGs based on degree and the maximum correntropy criterion (MCC) algorithms.

### Screening of prognosis-related genes and clinical correlation analysis

The GSE10846 dataset^25^, the GSE11318 dataset^26^, and the TCGA_DLBC databases were used to discover prognosis-related genes in DLBCL patients, and the clinical characteristics were extracted. For the investigation of the significant prognostic genes of ER stress-related DEGs, the samples of above the three datasets were divided into two groups based on the median value of differential ER stress-related DEGs expression and the intersection of them. The Kaplan–Meier (KM) curves were employed for survival analysis, and the log-rank method was used to compare the two groups, *P* < 0.05 was considered as significant difference. Simultaneously, the expression of ER stress-related DEGs in different COO and stages of DLBCL was analyzed by Kruskal Wallis test. In order to investigate the diagnostic role of ER stress-related DEGs in the GSE56315 dataset, the receiver operating characteristic (ROC) curve was drawn through the GSE56315 dataset using the pROC and plotROC packages^36^, and the area under the curve (AUC) was calculated. The experssion of ER stress-related DEGs in the TCGA_DLBC was also determined.

### Gene expression quantification on cultured cell lines

In this study, we detected gene expression in cultured cell lines, there are four cell lines including HBL-1 (A gift from Fujian Research Institute of Haematology, China), SUDHL2 (A gift from School of Medcine, Southeast Universtiy, China), SUDHL4 (Meisen, China) and GM12878 (BeNa, China) enrolled, thess cell lines were cultured in Roswell Park Memorial Institute 1640 (RPMI‐1640) medium (Gibco, US), supplemented with 10% fetal bovine serum (FBS, Gibco, US) and 1% Penicillin/Streptomycin (Gibco, US), and incubated at 37℃ in a 5% CO2 incubator. Total RNA were isolated using TRIzol reagent (Invitrogen, US), then HiScript® Q RT SuperMix for qPCR (Vazyme, China) was used to transcribe the RNA into cDNA. The Quantitative real‐time polymerase chain reaction (qRT-PCR) was performed on the CFX Connect Real-Time PCR Detection System (BioRad, US) with an HQ SYBR qPCR Mix (Without ROX) (Zomen, China). The mRNA expressions were quantified with the 2^−ΔΔCt^ method, and β-actin expression was used as an endogenous reference. The primer sequences of identified ER stress-related DEGs were purchased from Sunya, China (Supplementary Table S2).

### Immune microenvironment assessment

For evaluating the amount and proportion of the expression of ER stress-related DEGs in immune cells, CIBERSORTx^37,38^, a suite of machine learning tools that used the deconvolution algorithm to evaluate the proportion of various immune cells from the expression profiles of tumor tissues, and leukocyte signature matrix (LM22) feature matrices were used to estimate the proportion of immune cell in the DLBCL samples in the GSE10846 and GSE11318 datasets using the ESTIMATE package^39^, which provides the algorithm to calculate the tumor purity, stromal score, immune score.

### Construction and validation of the nomogram based on the prognostic model

A nomogram integrating the expression of ER stress-related DEGs and clinical characteristics of DLBCL patients, including tumor-node-metastasis (TNM) stage, Eastern Cooperative Oncology Group (ECOG) score, lactate dehydrogenase (LDH) ratio, and others, was constructed for prediction of prognosis in the GSE10846 as a training datasets. Furthermore, the nomogram’s prognosis prediction performance was confirmed in the GSE11318 datasets (validation dataset) by comparing the fitting degree between the observed and optimized values.

### Statistical analysis

R programming (version 4.1) was used for all statistical analysis. We used the Student’s t-test or one-way analysis of variance (ANOVA) to compare normally distributed continuous variables between groups and Mann–Whitney test or Kruskal–Wallis test to examine non-normally distributed continuous variables. Two-sided *P* values less than 0.05 indicated statistical significance.

## Results

### Screening of ER stress-related DEGs

**Figure 1:**
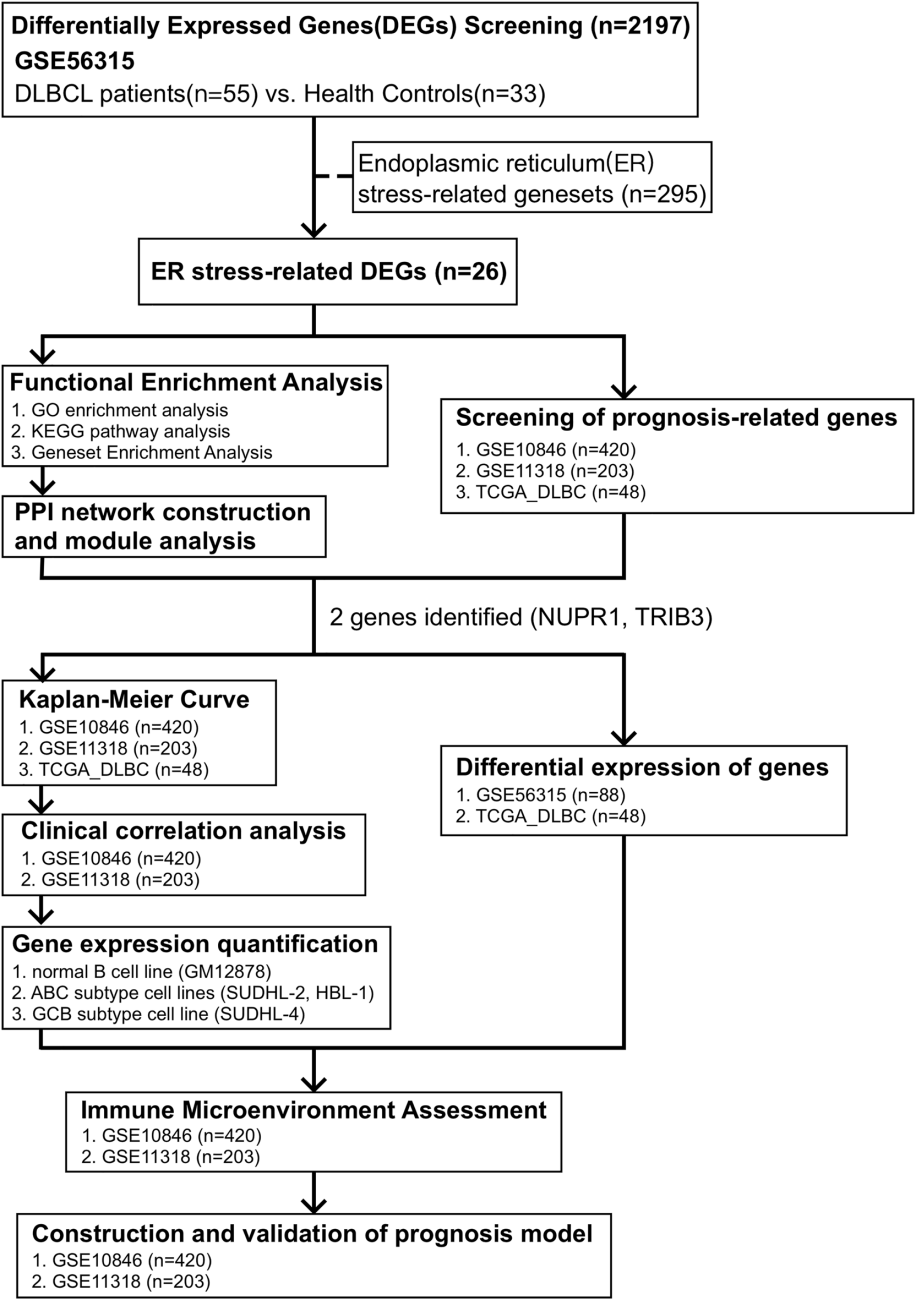
Workflow of this study.

**Figure 2:**
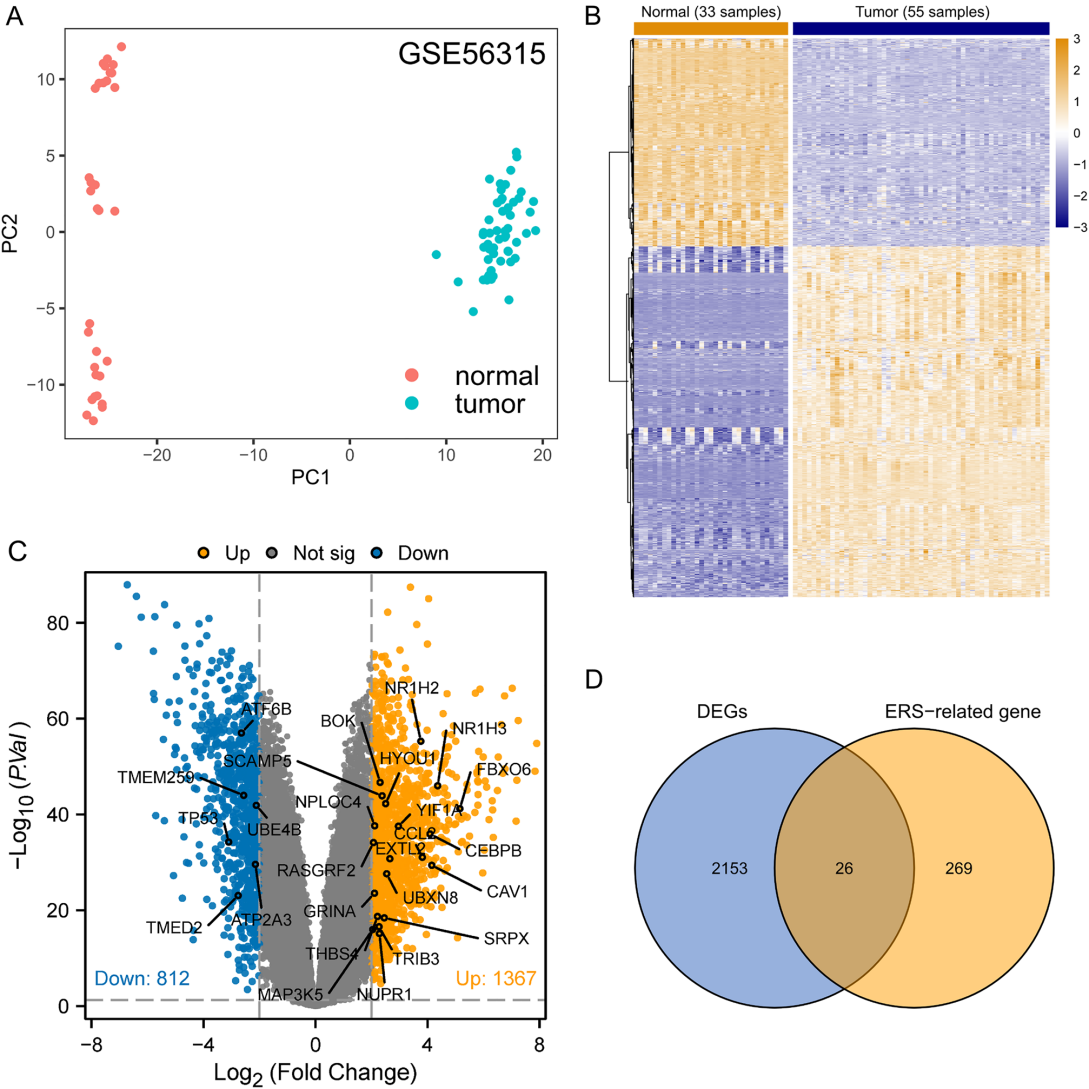
Identification of ER stress-related DEGs. (**A**) PCA show the dimensionality reduction distribution of DLBCL samples and NCs in the GSE56315 dataset. (**B**)–(**C**) Heatmap plot and volcano plot of DEGs in DLBCL samples vs. NCs based on the GSE56315 dataset. (**D**) Venn diagram of ER stress-related genes and DEGs. *DLBCL* Diffuse Large B Cell Lymphoma, *DEGs* differentially expressed genes, *ER* endoplasmic reticulum, *PCA* principal component analysis.

This study’s flowchart is depicted in Fig. 1. The clinical characteristics and standardized gene data were extracted from the GSE56315 dataset using GEOquery package^40^, and there are significant differences between DLBCL and NCs based on the principal component analysis (PCA), as shown in Fig. 2A. A total of 2179 DEGs were obtained through difference analysis using the limma package^28^, among which 1367 genes were upregulated and 812 genes were downregulated (Fig. 2B,C). After determining the overlap between ER stress-related genesets and DEGs through a Venn diagram, 26 ER stress-related DEGs were found (Fig. 2D).

### Functional enrichment analysis and PPI network construction

**Figure 3:**
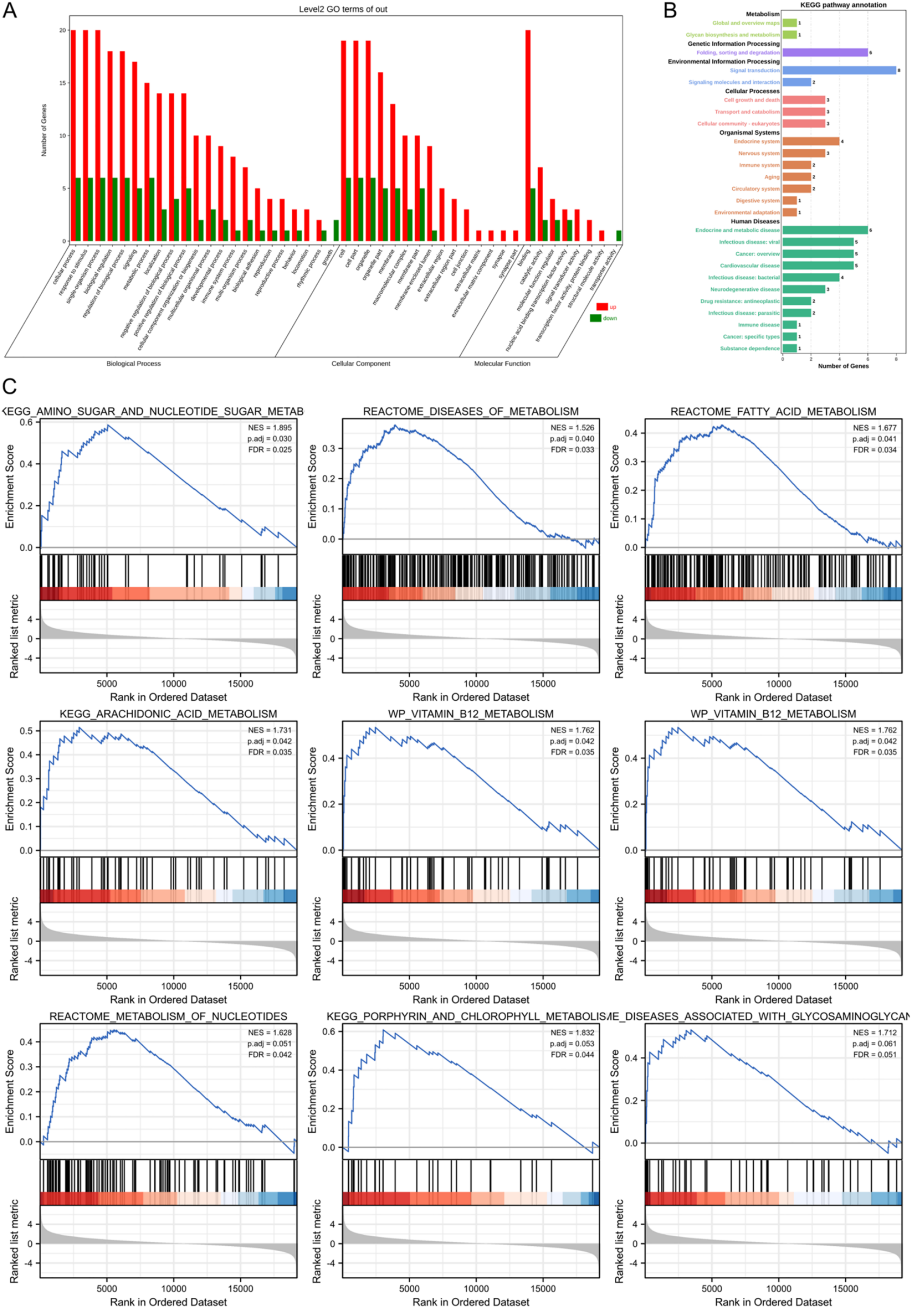
The functional enrichment analysis of ER stress-related DEGs. (**A**) GO enrichment analysis of ER stress-related DEGs. (**B**) KEGG pathway analysis of ER stress-related DEGs. (**C**) The enriched metabolic related pathways in DLBCL patients analyzed by GSEA. *DEGs* differentially expressed genes, *ER* endoplasmic reticulum, *GO* gene ontology, *GSEA* gene set enrichment analysis, *KEGG* kyoto encyclopedia of genes and genomes.

**Figure 4:**
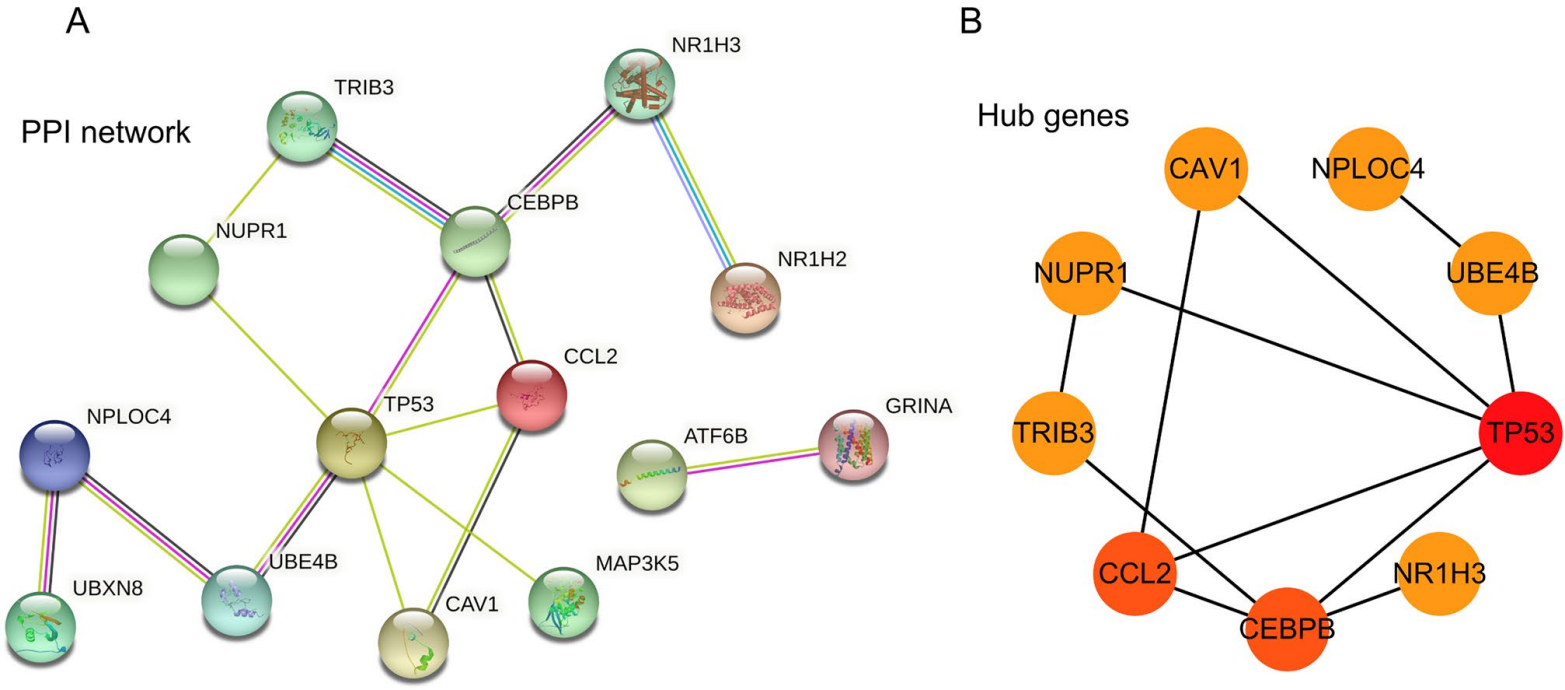
PPI network plot and hub genes of ER stress-related DEGs. (**A**) The PPI network of ER stress-related DEGs was visualized by Cytoscape software. (**B**) The hub genes were identified with the MCC score using cytoHubba plugin in Cytoscape software. *PPI* protein–protein interaction, *ER* endoplasmic reticulum, *DEGs* differentially expressed genes.

In order to find the enriched functions for the 26 ER stress-related DEGs, GO enrichment analysis were processed. The significantly enriched biological processes (BP) included response to ER stress, cellular response to stress, and response to stress. And the main top enrichment cellular component (CC) was ER (Fig. 3A). The KEGG pathway analysis showed that protein processing in ER, fluid shear stress, tumor necrosis factor (TNF) signaling pathway, atherosclerosis, and insulin resistance were enriched (Fig. 3B). More details of the top 50 significant items of GO enrichment analysis and KEGG pathway analysis (Ref: 231,102) could be shown in Supplementary Tables S3 and S4. The above results suggested that the metabolism and immune system were critical for DLBCL. We carried out a GSEA analysis to explore the metabolism and immune-related pathways; there were some pathways, including amino sugar and nucleotide sugar metabolism, diseases of metabolism reactome fatty acid metabolism, arachidonic acid metabolism, vitamin B12 metabolism were positively enriched in DLBCL patients (Fig. 3C). More details of the GSEA enrichment can be seen in Supplementary Table S5. For validation of the relationships among 26 ER stress-related DEGs, the STRING tool was used to assess with confidence (value ≥ 0.40), and there were 26 nodes and 15 edges with PPI network enrichment (Fig. 4A). The top ER stress-related DEGs based on MCC score were identified based on the cytoHubba plugin in Cytoscape software. There are 9 hub genes, including *TP53*, *CCL2*, *CEBPB*, *NUPR1*, *TRIB3*, *CAV1*, *UBE4B*, *NPLOC4*, and *NRIH3* (Fig. 4B), indicating they might play a significant role in DLBCL.

### Identification of significant prognostic gene

**Figure 5:**
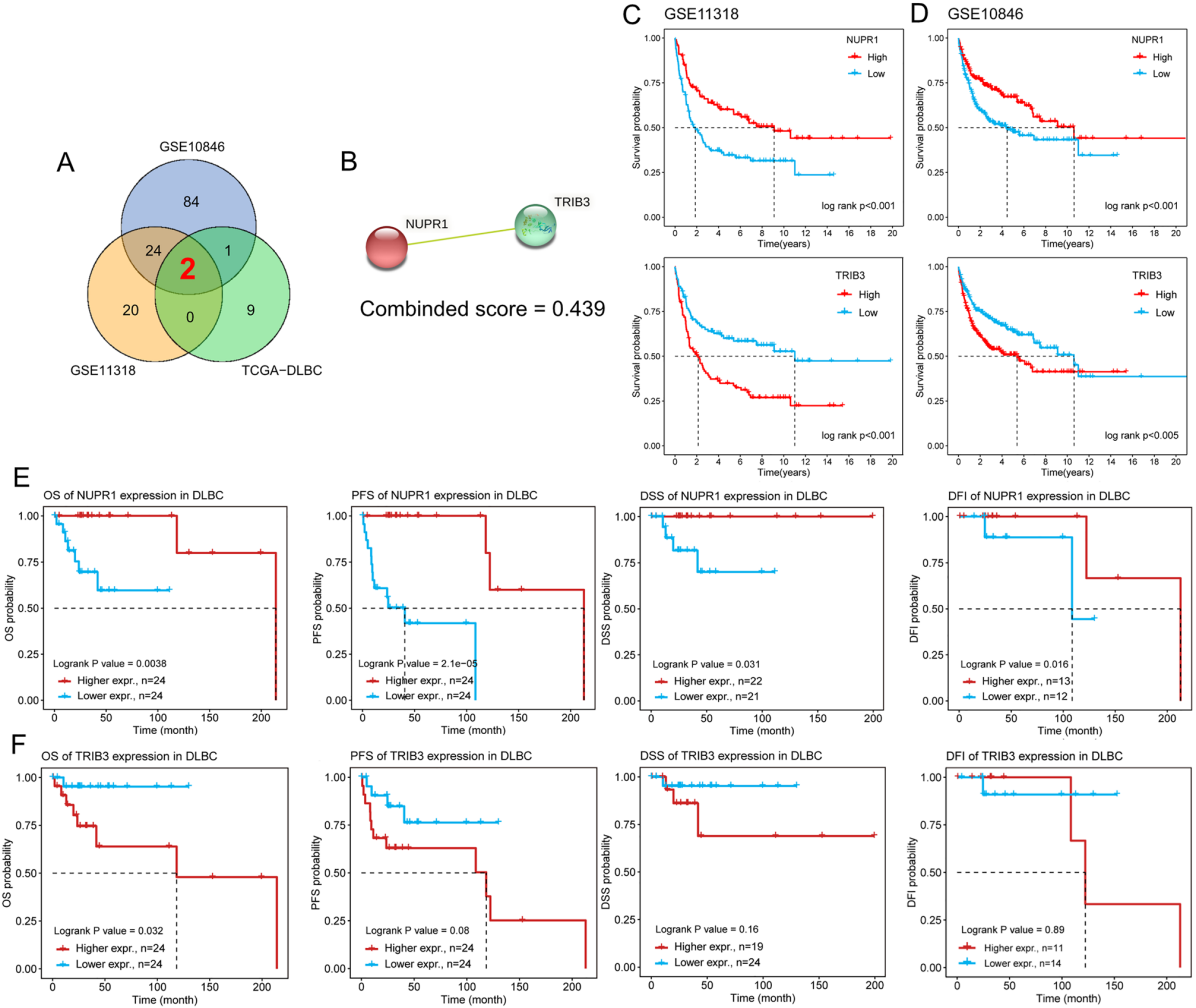
Prognosis-related genes and survival plot. (**A**) Venn diagram showed that 2 overlapped co-expressed prognosis-related genes were screened based on Log-rank test in the GSE10846, GSE11318, and TCGA_DLBC datasets. (**B**) Protein Interaction of *NUPR1* and *TRIB3* Genes. (**C**) The KM curves showed the OS of patients in the high-risk and low-risk groups in GSE10846 dataset. (**D**) The KM curves showed the OS of patients in the high-risk and low-risk groups in GSE11318 dataset. (**E**) The up-regulation *NUPR1* might be associated with better OS, PFS, DSS and DFI in TCGA_DLBC dataset. (**F**) The up-regulation TRIB3 might be associated with poor OS, PFS, DSS in TCGA_DLBC dataset. *DSS* disease specific survival; *DFI* disease free interval; *KM* Kaplan–Meier, *OS* over survival, *PFS* progression free survival, *TCGA* the cancer genome atlas.

To identify the significant ER stress-related genes and survival data in DLBCL patients, we analyzed the prognostic value of ER stress-related genes using the GEO datasets (GSE10846 and GSE11318) and the TCGA-DLBC dataset, which the clinical characteristics are shown in Table 1. It was found that *NUPR1* and *TRIB3* had survival differences in the above 3 datasets (Fig. 5A) and were among 26 ER stress-related DEGs. Interestingly, according to the previous PPI results, *NUPR1* and *TRIB3* genes might have a direct interaction, and the connection score was 0.439 (Fig. 5B). The KM curve showed that there was a significant difference between high and low expression of *NUPR1* and *TRIB3* (*P* < 0.01) in the GSE10846 and GSE11318 datasets (Fig. 5C,D). In the TCGA-DLBC dataset, we found that *NUPR1* and *TRIB3* not only have survival differences in over survival (OS), but also have survival differences in progress free survival (PFS), disease specific survival (DSS), and disease free interval (DFI), as shown in Fig. 5E,F. These results showed that patients with high expression of *NUPR1* and low expression of *TRIB3* might have a better prognosis.

**Table 1:** Clinical characteristics of GSE10846, GSE11318, and TCGA_DLBC.

Characteristic	levels	GSE10846	GSE11318	TCGA_DLBC
n		374	163	48
Event, n (%)	ALIVE	221 (59.1%)	67 (41.1%)	43 (89.58%)
	DEAD	153 (40.9%)	96 (58.9%)	5 (10.42%)
Gender, n (%)	Female	164 (43.9%)	73 (44.8%)	26 (54.17%)
	Male	210 (56.1%)	90 (55.2%)	22 (45.83%)
Age, n (%)	< 60	168 (44.9%)	64 (39.3%)	26 (54.17%)
	> = 60	206 (55.1%)	99 (60.7%)	22 (45.83%)
Fil microarray diagnosis, n (%)	ABC DLBCL	155 (41.4%)	70 (42.9%)	–
	GCB DLBCL	160 (42.8%)	66 (40.5%)	–
	Unclassified DLBCL	59 (15.8%)	27 (16.6%)	–
ECOG performance, n (%)	0	72 (20.5%)	34 (21.1%)	–
	1	190 (54%)	88 (54.7%)	–
	2	59 (16.8%)	28 (17.4%)	–
	3	26 (7.4%)	10 (6.2%)	–
	4	5 (1.4%)	1 (0.6%)	–
Stage, n (%)	I	60 (16.3%)	25 (15.4%)	8 (16.67%)
	II	109 (29.6%)	50 (30.9%)	17 (35.42%)
	III	85 (23.1%)	32 (19.8%)	5 (10.42%)
	IV	114 (31%)	55 (34%)	12 (25%)
Number of extranodal sites, n (%)	0	210 (61%)	134 (82.7%)	13 (27.08%)
	1	104 (30.2%)	28 (17.3%)	12 (25%)
	2	19 (5.5%)	–	6 (12.5%)
	3	8 (2.3%)	/	4 (8.33%)
	4	2 (0.6%)	–	1 (2.08%)
	5	1 (0.3%)	–	–
LDH ratio, median (IQR)		1.01 (0.77, 1.68)	1.02 (0.76, 1.68)	–

### Validation of the differential expression of *NUPR1*, *TRIB3*

**Figure 6:**
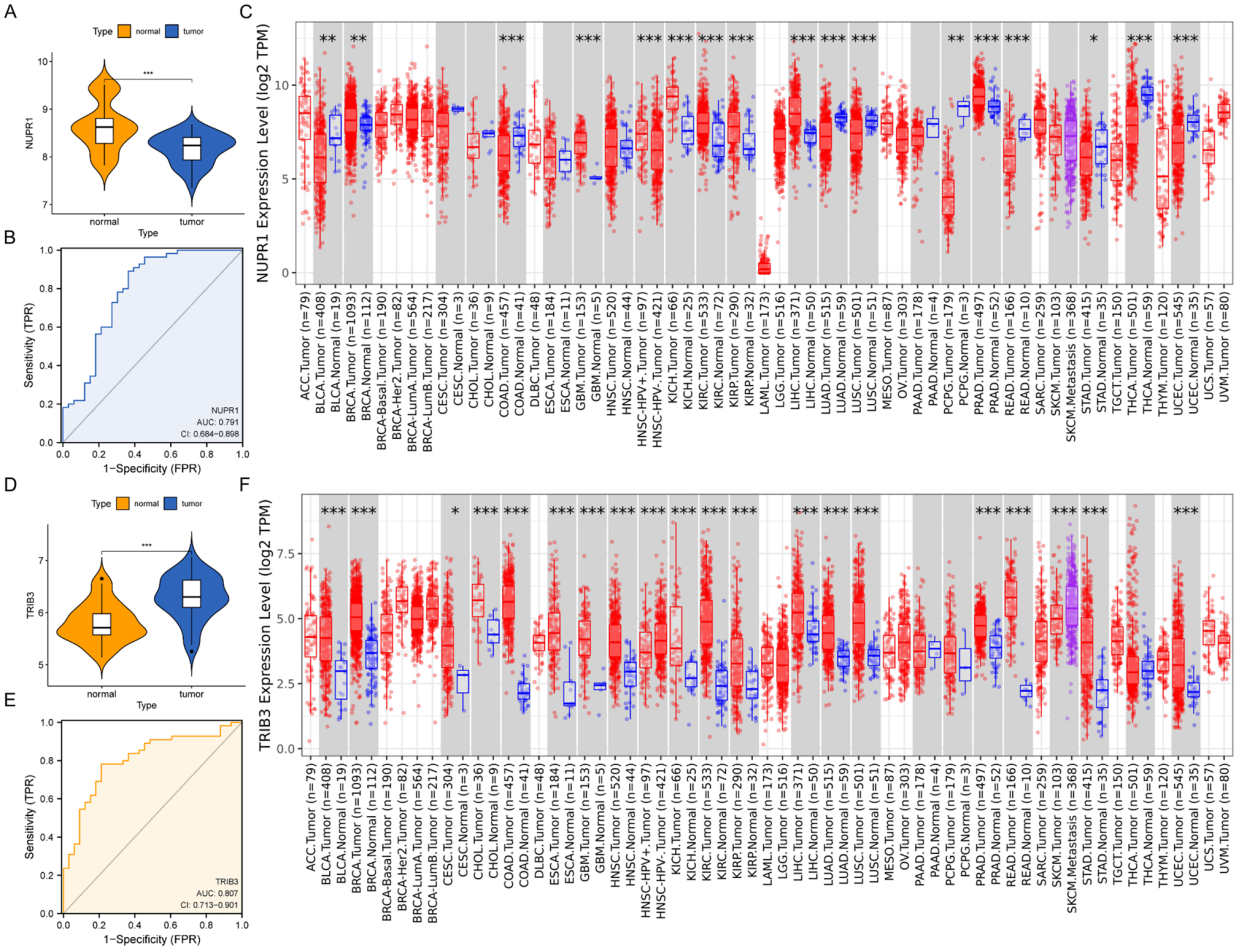
The identification of *NUPR1* and *TRIB3* in DLBCL. (**A**) Comparison of *NUPR1* expression level of DLBCL and NCs in GSE56315 dataset. (**B**) The ROC curves of *NUPR1* in GSE56315 dataset. (**C**) Pan-cancer analysis of *NUPR1* in cancer tissues and normal counterparts from TCGA-DLBC databases. D. Comparison of *TRIB3* expression level of DLBCL and NCs in GSE56315 dataset. (**E**) The ROC curves of *TRIB3* in GSE56315 dataset. (**F**) Pan-cancer analysis of *TRIB3* in TCGA databases. *DLBCL* diffuse large B cell lymphoma;* ROC receiver operating characteristic; TCGA* the cancer genome atlas.

We detected the expression of *NUPR1* and *TRIB3* in the GSE56315 dataset, the decreased expression of *NUPR1* and increased expression of *TRIB3* in DLBCL patients compared with NCs were showed, and the AUC was 0.791 and 0.807, respectively (Fig. 6A,B,D,E). Meanwhile, pan-cancer analysis based on the TIMER2 database^41^ (http://timer.cistrome.org) showed that *NUPR1* had an inconsistent expression trend in different tumors, the expression of *NUPR1* was decreased in bladder cancer, colon cancer, lung adenocarcinoma, prostate cancer, and other tumors. On the other hand, in breast cancer, renal clear cell carcinoma, and liver cancer, the expression of *NUPR1* was increased (Fig. 6C). The upregulated *TRIB3* might play an oncogenic role in tumors (Fig. 6F).

**Figure 7:**
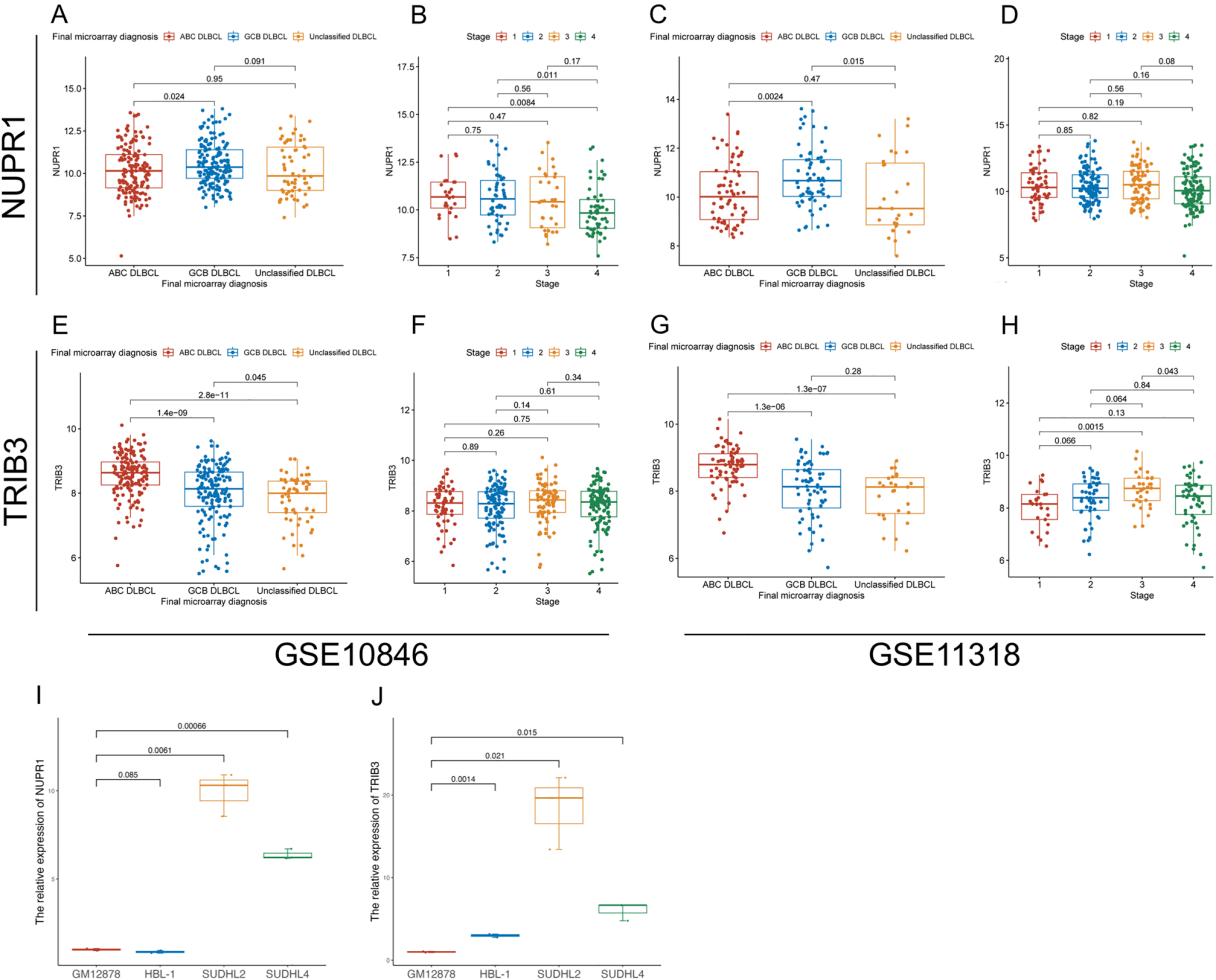
Differential expression of *NUPR1* and *TRIB3*. (**A**)–(**B**). The expression of *NUPR1* among different subtypes and stages in GSE10846 dataset; (**C**)–(**D**). The expression of *NUPR1* among different subtypes and stages in GSE11318 dataset; (**E**)–(**F**). The expression of *TRIB3* among different subtypes and stages in GSE10846 dataset; (**G**)–(**H**). The expression of *TRIB3* among different subtypes and stages in GSE11318 dataset; The qRT-PCR validation, (**I**) The expression of *NUPR1* of GM12878, HBL-1, SUDHL2 and SUDHL4; (**J**) The expression of *TRIB3* of GM12878, HBL-1, SUDHL2 and SUDHL4. * *P* < 0.05; ** *P* < 0.01; *** *P* < 0.001. qRT-PCR: quantitative real‐time polymerase chain reaction.

Next, the correlation of *NUPR1* and *TRIB3* with the clinical characteristics of the GSE10864 and GSE11318 datasets was confirmed. The COO of DLBCL often identified three subgroups including activated B-cell-like (ABC), germinal-center B-cell-like (GCB), and unclassified (UNC). As shown in Fig. 7, The expression of *NUPR1* in the ABC subtype was lower than in the GCB subtype both two GEO datasets (Fig. 7A,C), On the contrary, the expression of *TRIB3* in the ABC subtype was higher than GCB subtype (Fig. 7E,G). However, the expression of *NUPR1* and *TRIB3* in differential stages was not significantly different based on the two GEO datasets (Fig. 7B,D,F,H). It showed that downregulated *NUPR1* and upregulated *TRIB3* might be identified as vital biomarkers for DLBCL.

### The expression of *NUPR1* and *TRIB3* in cultured cell lines

We also detected the expression of *NUPR1* and *TRIB3* in different cell lines via qRT-PCR (Fig. 7I,J). The expression in *TRIB3* of DLBCL cell lines, including ABC subtype cell lines (HBL-1, SUDHL2) and GCB subtype cell lines (SUDHL4), were higher than normal B cell lines (GM12878) (*P* < 0.05), and ABC subtype cell lines were higher than GCB subtype (*P* < 0.05). But the expression of *NUPR1* of HBL-1 had no significant difference in comparison with GM12878 (*P* = 0.085), the expression of *NUPR1* in SUDHL2 and SUDHL4 was higher than GM12878 (*P* < 0.05). Both the expression of *NUPR1* and *TRIB3* in SUDHL2 than in SUDHL4 (*P* < 0.05).

### Relationship between the expression of *NUPR1*, *TRIB3* and immune infiltration

**Figure 8:**
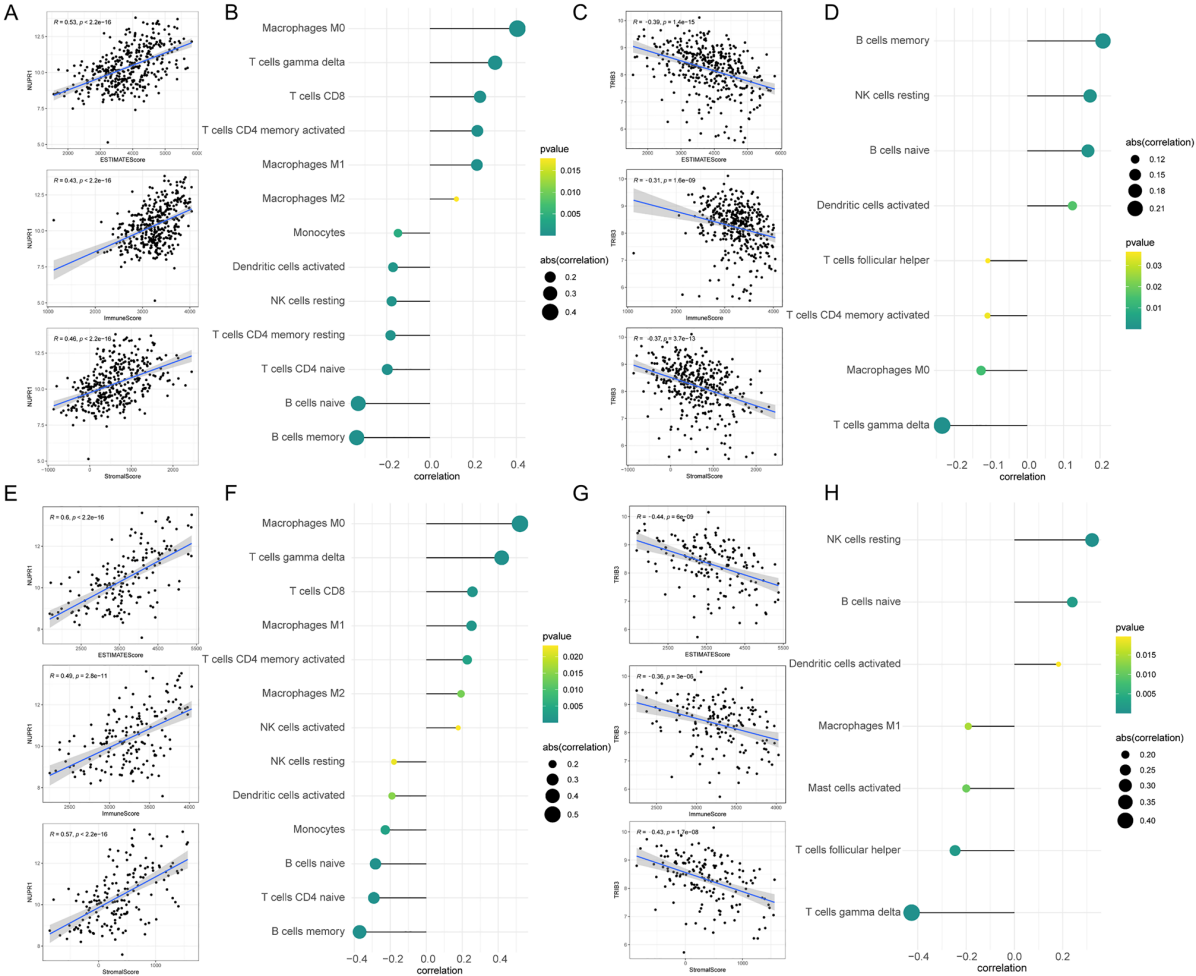
The correlation of prognostic genes with immune infiltration. (**A**) The relationship between the expression of *NUPR1* with ESTIMATE score, immune score and matrix score in the GSE10846 dataset. (**B**) The relationship between the expression of *NUPR1* and the different subsets of immune cell infiltrates in the GSE10846 dataset. (**C**) The relationship between the expression of *TRIB3* with ESTIMATE score, immune score and matrix score in the GSE10846 dataset. (**D**) The relationship between the expression of *TRIB3* and the different subsets of immune cell infiltrates in the GSE10846 dataset. (**E**) The relationship between the expression of *NUPR1* with ESTIMATE score, immune score and matrix score in the GSE11318 dataset. (**F**) The relationship between the expression of *NUPR1* and the different subsets of immune cell infiltrates in the GSE11318 dataset. (**G**) The relationship between the expression of *TRIB3* with ESTIMATE score, immune score and matrix score in the GSE11318 dataset. (**H**) The relationship between the expression of *TRIB3* and the different subsets of immune cell infiltrates in the GSE11318 dataset. |rho|> 0.4 and *P* < 0.05 were considered to be significantly correlated.

Based on our previous analysis, we speculated that *NUPR1* might inhibit the progress of DLBCL and *TRIB3* might promote it. Subsequently, the relationship between the expression of *NUPR1*, *TRIB3*, and immune infiltration was analyzed. As shown in Fig. 8, in the GSE10846 and GSE11318 datasets, *NUPR1* was positively correlated with the estimate score, immune score, and matrix score, simultaneously, the expression of *NUPR1* was positively correlated with M0 macrophages (Fig. 8A–D). However, *TRIB3* was significantly negatively correlated with the estimate score, immune score, and matrix score (Fig. 8E–H), and had no significance with all immune cells in both two GEO datasets. These results suggested that *NUPR1* might inhibit the progress of DLBCL by involving the immune response. But *TRIB3* had no close relationship with immune cells through correlation with tumor purity.

### Construction and validation of prognosis model

**Figure 9:**
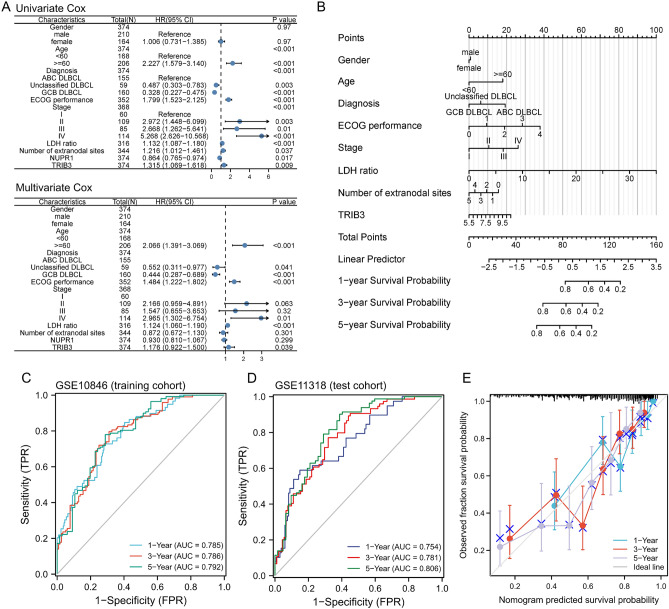
Construction and validation of prognosis model. (**A**) Forest plot summary of the univariate and multivariable analyses of *NUPR1*, *TRIB3* and other clinical characteristics. (**B**) Nomogram for predicting the probability of patient mortality at 1-, 3- and 5-year of OS. (**C**) The ROC for 1-, 2- and 3-year survival rate in the GSE10846 dataset, and the AUC was 0.785, 0.786 and 0.792, respectively. (**D**) The ROC for 1-, 2- and 3-year survival rate in the GSE11318 dataset, and the AUC was 0.754, 0.781 and 0.808, respectively. (**E**) 1-, 3-, and 5-year calibration curves of clinical prediction models for DLBCL patients, the model is validated by resampling with the bootstrap method, and the number of times was 1000. *AUC* area under curve, *DLBCL* diffuse large B cell lymphoma, *OS* overall survival.

Based on the GSE10846 and GSE11318 datasets, a clinical prognosis model was developed using *NUPR1* and *TRIB3* as characteristic variables and its accuracy and generalizability assessed. By combining *NUPR1* and *TRIB3* with other clinical characteristics of DLBCL patients, such as gender, age, diagnosis type, stage, LDH ratio, number of extranodal lymph nodes, and ECOG score. We found that the above risk factors were statistically different in univariate analysis except for gender. Subsequently, multivariate Cox analysis found that age (≥ 60 years old), stage, LDH ratio, and the expression of *TRIB3* were independent prognostic factors (Fig. 9A). Next, the nomogram for accurate patient prognosis and prediction was constructed based on the clinical characteristics of DLBCL patients and the expression of *TRIB3* (Fig. 9B). In order to evaluate the accuracy of the model, the time-dependent ROC curve was drawn using the GSE10846 dataset as the training cohort and the GSE11318 dataset as the test cohort. The AUC corresponding to 1, 3, and 5 years of both the training cohort and test cohort were all above 0.75 (Fig. 9C–D), indicating the nomogram model provided a good predictive accuracy of DLBCL. Furthermore, the calibration curve suggested that the consistency of the model is good at 1-, 3-, and 5-year intervals (Fig. 9E). These above results indicated that combining the clinical characteristics of DLBCL with the expression of *TRIB3* could predict the prognosis of patients with DLBCL in 1-, 3-, and 5-year intervals, which was expected to provide a more effective reference for clinicians and formulate effective intervention measures.

## Discussion

Due to differences in gene expression profiles and genetic alterations, DLBCL is a highly diverse lymphoid neoplasm that exhibits a wide range of clinical outcomes and therapeutic responses^2,3,9^. There has been significant development in targeted treatment and immunotherapy for DLBCL in recent years^3,5^, however, innovative treatment approaches and targets are urgently required. ER stress has emerged as a focal-point and forward position field in a variety of human malignancies during the last decade^22,23^, and is involved in many biological processes, such as apoptosis, autophagy^42^, ferroptosis^43^, and hypoxia^44^. But there is a lack of in-depth knowledge about the role of ER stress in the clinical progress of DLBCL.

So far as we know, our study is the first research on the relationship between the ER stress-related gene and DLBCL. These findings should help researchers in the future learn more about how to predict prognosis and treat DLBCL patients individually in the clinic. Based on the outcomes of previous bioinformatic analysis, we have found nine genes may play essential roles in DLBCL. As previously described^45–47^, it had been proven that *TP53, CCL2,* and *CEBPB* were involved in the carcinogenesis and development of DLBCL. *TP53* has been identified as one of the most frequently mutated genes and could be a valuable prognostic biomarker in both ABC and GCB DLBCL patients^45,46^. It was found that CEBPB and BCL2A1 can induce cell transformation and increase the survival of anaplastic large cell lymphomas cells^47^. However, no research has been reported about the role of *NUPR1*, *TRIB3*, *CAV1*, *UBE4B*, *NPLOC4*, and *NRIH3* in DLBCL. After identifying the prognostic value of ER stress-related genes using three datasets, *NUPR1* and *TRIB3* may play important roles in DLBCL carcinogenesis and development. NUPR1 is located in the nucleus of various cells, including cancer cells^48,49^. Borrello et al. demonstrated the essential role of NUPR1 which participated in the regulation of UPR and more broadly in the integrated stress response by interacting with eIF2α, and protected the liver from metabolic distress by controlling lipid homeostasis^50^. Howerer, Liu et al. found that NUPR1 was upregulated in the bone marrow of patients with multiple myeloma (MM)^51^, downregulation of NUPR1 might significantly inhibit cell proliferation and promote autophagy-mediated apoptosis in MM^52^. NUPR1 could also active autophagy and bind to the promoter regions of some autophagy-related genes, such as BECN1, GREB1, RAB31, PGR, CYP1B1, thereby regulating breast cancer metastasis and transcription^49,53^. Histone methyltransferase Dot1L might inhibit pancreatic cancer cell apoptosis by targeting NUPR1, and overexpressed NUPR1 also inhibited pancreatic cancer cell apoptosis^54^. In summary, NUPR1 can play an important role in cell stress and stress‑related apoptosis^48,55^. We found the level of *NUPR1* in DLBCL patients to be downregulated, and that in the GCB subtype was significantly higher than the ABC subtype in our study. We also found *NUPR1* could involve in the immune response.

Compared with noncancerous tissues, *TRIB3* expression was markedly increased in DLBCL patients, particularly in the ABC subtype and later stages, suggesting that *TRIB3* may play a carcinogenic role in DLBCL^56–59^. As a member of the mammalian pseudokinase tribbles family, TRIB3 can interfere with a lot of proteins, such as kinase-dependent proteins, transcription factors, and ubiquitin ligases^60,61^. Evidences show that TRIB3 can regulate the downstream biological process of ER stress, alleviate cell stress, and promote cell survival^58,62,63^. Ohoka et al. demonstrated that certain ER stress inducers, including low glucose and hypoxia, may increase *TRIB3* expression^62–64^. Twist Family BHLH Transcription Factor 1 (TWIST1) is stabilized in part by *TRIB3* binding to its WR domain and blocking its ubiquitination, depletion of *TRIB3* can boost TWIST1 degradation and increase sensitivity to all-trans retinoic acid (ATRA), consistent with its role in regulating carcinogenesis and progression^63^. *TRIB3* overexpression in human gastric cancer was related to tumor angiogenesis and a poor prognosis^65^. Through analysis of lymphoma specimens, TRIB3 expression was positively correlated with MYC expression^66^, MYC is a transcription factor, and its alterations have been considered to be associated with aggressive clinical behavior in DLBCL^67^. Mechanistically, E3 ubiquitin ligase UBE3B-mediated MYC ubiquitination and degradation can be inhibited by TRIB3 bind to MYC^66^. These results are in agreement with our studies and imply that *TRIB3* may be a critical regulatory factor in driving cancer cell proliferation, migration, and invasion^57,58,68^. Furthermore, our bioinformatic analysis indicated that *TRIB3* could be a significant survival predictor and a potential therapeutic target for DLBCL.

Our bioinformatic analysis suggested a direct connection between *NUPR1* and *TRIB3*. Methamphetamine (METH) exposed to rat and PC12 cell lines could increase the expression of NUPR1 accompanied by CHOP and TRIB3 upregulation and promote apoptosis and autophagy^69^, suggesting the NUPR1/CHOP/TRIB3 signal pathway plays a crucial function in the regulation of apoptosis and autophagy via ER stress^69,70^. There were shown the expression of *NUPR1* and *TRIB3* concurrently. Unfortunately, our study suggested that both the lower expression of *NUPR1* and the higher expression of *TRIB3* meant a worse clinical prognosis for DLBCL patients. Furthermore, the qRT-PCR test showed the expression of *TRIB3* in HBL-1, SUDHL2, and SUDHL4 was higher than GM12878, and these results were consistent with our bioinformatic analysis, However, the expression of *NUPR1* was not compatible with these analysis.

The clinical prognostic model was built based on the GSE10846 and GSE11318 datasets, using NUPR1 and TRIB3 as feature variables, and evaluated for accuracy and generalizability. There are some authors^71,72^ who have built the prognosis model that is similar to ours. Lv et al. showed that carcinoembryonic antigen, N stage, and surgical method were independent prognostic factors for overall survival in patients with obstructive colorectal cancer using a nomogram model^71^. For researching the role of ACK1-associated immunomodulators in non-small cell lung cancer (NSCLC), Zhu J et al. Established a multiple-gene risk prediction model, and the results showed the risk scores were an independent prognosis predictor in the TCGA lung cohorts^72^. It was suggested that ACK1 may be a potential immunotherapeutic target^72^. Numerous types of tumors may share similar genetic or molecular features, there are a lot of studies^48,55,57,60,61^ showed that *NUPR1* and *TRIB3* are involved in cancer biology, and we think this prognosis model that includes these genes may be applied to more types of tumors. However, due to the specificity and complexity of different types of cancer, further studies should verify and adjust the accuracy and reliability of these identical markers and explore the relationship between their biological functions and clinical applications in numerous types of tumors.

Some limitations existed in this study. First, the prognostic model was built and validated using publicly available data; hence, more prospective studies are needed to confirm its accuracy and utility. Second, by narrowing our focus to only two ER stress-related genes, *NUPR1* and *TRIB3*, we may have missed other noteworthy prognostic genes for DLBCL. Third, only four cell lines were used to verify the expression of *NUPR1* and *TRIB3,* and we will carry out more cell lines for research if the experimental conditions are confirmed.

## Conclusion

In summary, our study clarified that downregulation the expression of *NUPR1* and upregulation of *TRIB3* in DLBCL patients. Further study has shown *NUPR1* may inhibit the progress of DLBCL by involving the immune response, and *TRIB3* should be a carcinogenic gene in DLBCL through regulating ER stress. Moreover, we found that combining the clinical characteristics of DLBCL by *TRIB3* expression could better predict the prognosis of DLBCL patients, suggesting *TRIB3* might serve as a potential therapeutic target and prognostic factor in DLBCL.

### Supplementary Information

Supplementary Tables.

## Data Availability

All of datasets used in this study can be found in online repositories. The names of the repository/repositories and accession number(s) can be found in the article/Supplementary Material.
