# Supplementary material for: Analysis of endoplasmic reticulum stress-related gene signature for the prognosis and pattern in diffuse large B cell lymphoma
Source: Sci Rep. 2023 Aug 25;13:13894. doi: 10.1038/s41598-023-38568-x (PMC10457392; doi:10.1038/s41598-023-38568-x)
Supplement: Supplementary file 1 — Supplementary Tables. [file 41598_2023_38568_MOESM1_ESM.docx]

# Supplementary

Table S1. Basical data of this study.

| datasets | platform | type | samples(all) |
| --- | --- | --- | --- |
| GSE56315 | GPL570 | Expression profiling by array | 88 |
| GSE10846 | GPL570 | Expression profiling by array | 420 |
| GSE11318 | GPL570 | Expression profiling by array | 203 |
| TCGA-DLBC | Illumina | high throughput sequencing | 48 |

Table S2. Primer of gene symbol

| Gene symbol | primer |
| --- | --- |
| *NUPR1* | F: 5’-GGAAAGGTCGCACCAAGAGAG-3’ |
|  | R: 5’-CTCTCTGAATTCTGCAGCTTGG-3’ |
| *TRIB3* | F: 5’-GAGATACTCAGCTCACGGGC-3’ |
|  | R: 5’-ATCTTGCCGAAGAGCAGGAC-3’ |
| *Actin* | F: 5’-TGACGTGGACATCCGCAAAG-3’ |
|  | R: 5’-CTGGAAGGTGGACAGCGAGG-3’ |

Table S3 GO enrichment analysis (Top50)

| class | id | Descrption | Pvalue | Qvalue |
| --- | --- | --- | --- | --- |
| BP | GO:0034976 response to endoplasmic reticulum stress | response to endoplasmic reticulum stress | 3.40E-44 | 6.95E-41 |
| BP | GO:0033554 cellular response to stress | cellular response to stress | 2.40E-24 | 2.44E-21 |
| BP | GO:0006950 response to stress | response to stress | 1.38E-16 | 9.40E-14 |
| BP | GO:0070059 intrinsic apoptotic signaling pathway in response to endoplasmic reticulum stress | intrinsic apoptotic signaling pathway in response to endoplasmic reticulum stress | 2.39E-14 | 1.22E-11 |
| CC | GO:0005783 endoplasmic reticulum | endoplasmic reticulum | 2.22E-12 | 4.75E-10 |
| BP | GO:0006986 response to unfolded protein | response to unfolded protein | 2.94E-12 | 1.20E-09 |
| BP | GO:0097193 intrinsic apoptotic signaling pathway | intrinsic apoptotic signaling pathway | 4.80E-12 | 1.63E-09 |
| BP | GO:0035966 response to topologically incorrect protein | response to topologically incorrect protein | 8.56E-12 | 2.50E-09 |
| BP | GO:0097190 apoptotic signaling pathway | apoptotic signaling pathway | 1.30E-11 | 3.31E-09 |
| BP | GO:0080135 regulation of cellular response to stress | regulation of cellular response to stress | 7.49E-11 | 1.70E-08 |
| BP | GO:1903573 negative regulation of response to endoplasmic reticulum stress | negative regulation of response to endoplasmic reticulum stress | 1.32E-10 | 2.69E-08 |
| BP | GO:0010033 response to organic substance | response to organic substance | 2.06E-10 | 3.83E-08 |
| BP | GO:0051716 cellular response to stimulus | cellular response to stimulus | 3.17E-10 | 5.39E-08 |
| BP | GO:0030968 endoplasmic reticulum unfolded protein response | endoplasmic reticulum unfolded protein response | 4.82E-10 | 7.57E-08 |
| CC | GO:0044432 endoplasmic reticulum part | endoplasmic reticulum part | 8.42E-10 | 9.01E-08 |
| BP | GO:0034620 cellular response to unfolded protein | cellular response to unfolded protein | 1.41E-09 | 2.06E-07 |
| BP | GO:0035967 cellular response to topologically incorrect protein | cellular response to topologically incorrect protein | 3.57E-09 | 4.86E-07 |
| CC | GO:0005789 endoplasmic reticulum membrane | endoplasmic reticulum membrane | 1.07E-08 | 7.10E-07 |
| CC | GO:0042175 nuclear outer membrane-endoplasmic reticulum membrane network | nuclear outer membrane-endoplasmic reticulum membrane network | 1.33E-08 | 7.10E-07 |
| BP | GO:0036503 ERAD pathway | ERAD pathway | 8.29E-09 | 1.06E-06 |
| BP | GO:0042221 response to chemical | response to chemical | 1.03E-08 | 1.19E-06 |
| BP | GO:1901214 regulation of neuron death | regulation of neuron death | 1.05E-08 | 1.19E-06 |
| BP | GO:0048550 negative regulation of pinocytosis | negative regulation of pinocytosis | 1.12E-08 | 1.20E-06 |
| BP | GO:0006984 ER-nucleus signaling pathway | ER-nucleus signaling pathway | 1.24E-08 | 1.26E-06 |
| BP | GO:0070997 neuron death | neuron death | 2.56E-08 | 2.49E-06 |
| BP | GO:0050896 response to stimulus | response to stimulus | 4.13E-08 | 3.83E-06 |
| BP | GO:0008219 cell death | cell death | 5.81E-08 | 4.94E-06 |
| BP | GO:0016265 death | death | 5.81E-08 | 4.94E-06 |
| BP | GO:0080134 regulation of response to stress | regulation of response to stress | 7.00E-08 | 5.72E-06 |
| BP | GO:0006915 apoptotic process | apoptotic process | 9.00E-08 | 7.07E-06 |
| BP | GO:2001242 regulation of intrinsic apoptotic signaling pathway | regulation of intrinsic apoptotic signaling pathway | 9.41E-08 | 7.12E-06 |
| BP | GO:0030433 ER-associated ubiquitin-dependent protein catabolic process | ER-associated ubiquitin-dependent protein catabolic process | 1.54E-07 | 1.12E-05 |
| BP | GO:0010941 regulation of cell death | regulation of cell death | 1.70E-07 | 1.20E-05 |
| BP | GO:0043065 positive regulation of apoptotic process | positive regulation of apoptotic process | 1.87E-07 | 1.28E-05 |
| BP | GO:0043068 positive regulation of programmed cell death | positive regulation of programmed cell death | 2.03E-07 | 1.34E-05 |
| BP | GO:0012501 programmed cell death | programmed cell death | 2.23E-07 | 1.42E-05 |
| BP | GO:0048548 regulation of pinocytosis | regulation of pinocytosis | 2.34E-07 | 1.45E-05 |
| CC | GO:0012505 endomembrane system | endomembrane system | 3.59E-07 | 1.54E-05 |
| BP | GO:0007165 signal transduction | signal transduction | 2.67E-07 | 1.61E-05 |
| BP | GO:0010942 positive regulation of cell death | positive regulation of cell death | 4.23E-07 | 2.46E-05 |
| BP | GO:0043523 regulation of neuron apoptotic process | regulation of neuron apoptotic process | 4.47E-07 | 2.47E-05 |
| BP | GO:0048583 regulation of response to stimulus | regulation of response to stimulus | 4.48E-07 | 2.47E-05 |
| BP | GO:0042981 regulation of apoptotic process | regulation of apoptotic process | 5.14E-07 | 2.76E-05 |
| BP | GO:0048585 negative regulation of response to stimulus | negative regulation of response to stimulus | 5.27E-07 | 2.76E-05 |
| BP | GO:1990440 positive regulation of transcription from RNA polymerase II promoter in response to endoplasmic reticulum stress | positive regulation of transcription from RNA polymerase II promoter in response to endoplasmic reticulum stress | 6.11E-07 | 3.07E-05 |
| BP | GO:0043067 regulation of programmed cell death | regulation of programmed cell death | 6.17E-07 | 3.07E-05 |
| BP | GO:0023051 regulation of signaling | regulation of signaling | 8.04E-07 | 3.91E-05 |
| BP | GO:0006508 proteolysis | proteolysis | 9.32E-07 | 4.42E-05 |
| BP | GO:0009966 regulation of signal transduction | regulation of signal transduction | 9.61E-07 | 4.46E-05 |
| BP | GO:0051402 neuron apoptotic process | neuron apoptotic process | 9.88E-07 | 4.48E-05 |

note: BP, Biological Process; CC, cellular component.

Table S4. KEGG pathway analysis (Top50)

| class | ID | Descrption | Pvalue |
| --- | --- | --- | --- |
| Genetic Information Processing | ko04141 | Protein processing in endoplasmic reticulum | 1.37E-06 |
| Environmental Information Processing | ko04668 | TNF signaling pathway | 1.12E-04 |
| Human Diseases | ko05418 | Fluid shear stress and atherosclerosis | 2.61E-04 |
| Human Diseases | ko04931 | Insulin resistance | 2.08E-03 |
| Human Diseases | ko05144 | Malaria | 6.86E-03 |
| Human Diseases | ko05014 | Amyotrophic lateral sclerosis | 7.60E-03 |
| Human Diseases | ko01524 | Platinum drug resistance | 1.25E-02 |
| Human Diseases | ko05163 | Human cytomegalovirus infection | 1.54E-02 |
| Organismal Systems | ko04211 | Longevity regulating pathway | 1.95E-02 |
| Organismal Systems | ko04657 | IL-17 signaling pathway | 2.03E-02 |
| Human Diseases | ko05017 | Spinocerebellar ataxia | 2.15E-02 |
| Human Diseases | ko05202 | Transcriptional misregulation in cancer | 2.20E-02 |
| Environmental Information Processing | ko04010 | MAPK signaling pathway | 3.05E-02 |
| Environmental Information Processing | ko04071 | Sphingolipid signaling pathway | 3.26E-02 |
| Organismal Systems | ko04722 | Neurotrophin signaling pathway | 3.30E-02 |
| Organismal Systems | ko04919 | Thyroid hormone signaling pathway | 3.40E-02 |
| Cellular Processes | ko04210 | Apoptosis | 4.11E-02 |
| Organismal Systems | ko04261 | Adrenergic signaling in cardiomyocytes | 4.65E-02 |

Table S5. GSEA enrichment

| Description | NES | pvalue | FDR |
| --- | --- | --- | --- |
| KEGG_AMINO_SUGAR_AND_NUCLEOTIDE_SUGAR_METABOLISM | 1.89 | 1.34E-03 | 2.53E-02 |
| REACTOME_DISEASES_OF_METABOLISM | 1.53 | 2.20E-03 | 3.33E-02 |
| REACTOME_FATTY_ACID_METABOLISM | 1.68 | 2.28E-03 | 3.41E-02 |
| KEGG_ARACHIDONIC_ACID_METABOLISM | 1.73 | 2.59E-03 | 3.54E-02 |
| WP_VITAMIN_B12_METABOLISM | 1.76 | 2.67E-03 | 3.54E-02 |
| KEGG_ETHER_LIPID_METABOLISM | 1.87 | 2.92E-03 | 3.59E-02 |
| REACTOME_METABOLISM_OF_NUCLEOTIDES | 1.63 | 3.74E-03 | 4.23E-02 |
| KEGG_PORPHYRIN_AND_CHLOROPHYLL_METABOLISM | 1.83 | 4.21E-03 | 4.43E-02 |
| REACTOME_DISEASES_ASSOCIATED_WITH_GLYCOSAMINOGLYCAN_METABOLISM | 1.71 | 5.33E-03 | 5.06E-02 |
| REACTOME_PEROXISOMAL_LIPID_METABOLISM | 1.74 | 5.61E-03 | 5.28E-02 |
| WP_AMINO_ACID_METABOLISM | 1.58 | 8.67E-03 | 6.87E-02 |
| REACTOME_METABOLISM_OF_VITAMINS_AND_COFACTORS | 1.46 | 8.93E-03 | 7.02E-02 |
| WP_PURINE_METABOLISM | 1.67 | 9.54E-03 | 7.32E-02 |
| REACTOME_METABOLISM_OF_PORPHYRINS | 1.70 | 1.17E-02 | 8.37E-02 |
| WP_FOLATE_METABOLISM | 1.53 | 1.80E-02 | 1.11E-01 |
| WP_PURINE_METABOLISM_AND_RELATED_DISORDERS | 1.64 | 1.95E-02 | 1.16E-01 |
| REACTOME_PHOSPHOLIPID_METABOLISM | 1.35 | 1.99E-02 | 1.17E-01 |
| WP_EICOSANOID_METABOLISM_VIA_CYCLO_OXYGENASES_COX | 1.61 | 2.00E-02 | 1.18E-01 |
| REACTOME_METABOLISM_OF_CARBOHYDRATES | 1.32 | 2.12E-02 | 1.22E-01 |
| REACTOME_GLYCOSAMINOGLYCAN_METABOLISM | 1.40 | 2.14E-02 | 1.22E-01 |
| KEGG_GLYOXYLATE_AND_DICARBOXYLATE_METABOLISM | 1.63 | 2.33E-02 | 1.27E-01 |
| KEGG_PROPANOATE_METABOLISM | 1.53 | 2.79E-02 | 1.42E-01 |
| KEGG_DRUG_METABOLISM_OTHER_ENZYMES | 1.52 | 3.18E-02 | 1.53E-01 |
| REACTOME_METABOLISM_OF_ANGIOTENSINOGEN_TO_ANGIOTENSINS | 1.56 | 3.22E-02 | 1.54E-01 |
| KEGG_STARCH_AND_SUCROSE_METABOLISM | 1.51 | 3.44E-02 | 1.59E-01 |
| KEGG_PYRIMIDINE_METABOLISM | 1.39 | 3.49E-02 | 1.61E-01 |
| REACTOME_ARACHIDONIC_ACID_METABOLISM | 1.45 | 3.86E-02 | 1.71E-01 |
| REACTOME_DEFECTS_IN_COBALAMIN_B12_METABOLISM | 1.52 | 4.13E-02 | 1.78E-01 |
| WP_NUCLEOTIDE_METABOLISM | 1.54 | 4.29E-02 | 1.82E-01 |
| KEGG_ALPHA_LINOLENIC_ACID_METABOLISM | 1.52 | 4.30E-02 | 1.82E-01 |
| WP_IRON_METABOLISM_IN_PLACENTA | 1.54 | 4.34E-02 | 1.82E-01 |
| KEGG_GLYCEROPHOSPHOLIPID_METABOLISM | 1.42 | 4.38E-02 | 1.83E-01 |
| KEGG_RETINOL_METABOLISM | 1.45 | 4.48E-02 | 1.86E-01 |
| REACTOME_METABOLISM_OF_STEROID_HORMONES | 1.48 | 4.50E-02 | 1.87E-01 |
| REACTOME_METABOLISM_OF_WATER_SOLUBLE_VITAMINS_AND_COFACTORS | 1.33 | 4.77E-02 | 1.92E-01 |
| WP_PYRIMIDINE_METABOLISM | 1.38 | 4.77E-02 | 1.92E-01 |
| KEGG_GLUTATHIONE_METABOLISM | 1.41 | 5.33E-02 | 2.04E-01 |
| REACTOME_METABOLISM_OF_STEROIDS | 1.32 | 5.52E-02 | 2.10E-01 |
| REACTOME_METABOLISM_OF_FAT_SOLUBLE_VITAMINS | 1.39 | 6.01E-02 | 2.23E-01 |
| REACTOME_SULFUR_AMINO_ACID_METABOLISM | 1.45 | 6.41E-02 | 2.35E-01 |
| KEGG_PURINE_METABOLISM | 1.29 | 6.54E-02 | 2.37E-01 |
| REACTOME_VITAMIN_D_CALCIFEROL_METABOLISM | 1.48 | 7.10E-02 | 2.49E-01 |
| REACTOME_BILE_ACID_AND_BILE_SALT_METABOLISM | 1.38 | 7.11E-02 | 2.49E-01 |
